# Gut microbiota deficiency ameliorates multiple myeloma and myeloma-related bone disease by Th17 cells in mice models

**DOI:** 10.7150/jca.88799

**Published:** 2023-09-25

**Authors:** Jia Liu, Fang Xie, Zhi-gang Yi, Tao Ma, Wen-ting Tie, Yan-hong Li, Jun Bai, Lian-sheng Zhang

**Affiliations:** Department of Hematology, Lanzhou University Second Hospital, Lanzhou 730000, China.

**Keywords:** Multiple myeloma, Multiple myeloma bone disease, Gut microbiome, 16S rRNA sequencing, IL-17/Th17 cell, Bone resorption

## Abstract

**Purpose:** Multiple myeloma, the second most common hematological tumor, is currently incurable. Multiple myeloma-related bone disease is a characteristic clinical symptom that seriously affects the survival and prognosis of patients. In recent years, gut microbiota has been shown to play an important role in the occurrence and development of multiple myeloma. However, whether and how it affects the development of myelomatous bone disease is unclear.

**Methods:** To investigate the mechanism and influence of the microbiota on multiple myeloma and myeloma bone disease, a myeloma-gut microbiota deletion mice model was established. 16S rRNA sequencing was used to analysis of bacterial flora changes. Histochemical staining and bone micro-CT were used to assess the severity of bone disease. Bone marrow tumor load and spleen Th17 cells were detected by flow cytometry.

**Results:** Histochemical staining revealed a reduced tumor burden after eliminating gut microbial communities in mice by administering a mixture of antibiotics. According to the 16S rRNA sequencing of intestinal contents, antibiotic treatment resulted in a significant change in the microbiota of the mice. Bone micro-CT demonstrated that antibiotic treatment could reduce bone lesions caused by myeloma while increasing mineral density, bone volume fraction, trabecular bone thickness, and trabecular number. Meanwhile, histochemical staining of the bone found that the enhanced bone resorption was weakened by the change of flora. These results were consistent with the concentration of IL17 in serum and the frequency of Th17 cells in spleen.

**Conclusions:** Herein, the effects of the gut microbiome on myeloma bone disease are investigated for the first time, providing new insight into its pathogenesis and suggesting that gut microbiota may serve as a therapeutic target in multiple myeloma-associated bone diseases.

## Introduction

Multiple myeloma (MM) is the second most common hematological malignancy and the most common primary bone malignancy 1. Multiple myeloma-related bone disease (MMBD) has severe consequences, such as severe bone loss, bone pain, and pathological fractures, which can significantly reduce patients' quality of life and affect their prognosis. MMBD is caused by excessive osteoclast (OC) resorption and persistent inhibition of osteoblastic (OB) bone formation, resulting in incurable lytic lesions even when patients are in complete and prolonged remission 2-4. Myeloma bone disease is characterized by increased osteoclast activity, decreased osteoblast activity, and disruption of the bone marrow (BM) microenvironment. Current treatment strategies for myeloma bone disease revolve mainly around bisphosphonates, which can be absorbed by OC during bone remodeling, impairing OC production and destroying OC activity 5. Studies have shown that bone turnover markers C-terminal telopeptide of type I collagen (CTX-1) in myeloma patients are elevated in newly diagnosed patients 6. Importantly, since bisphosphonates are excreted by the kidneys, they should not be used in patients with renal insufficiency. Denosumab, a monoclonal antibody that can specifically bind to Receptor Activator for Nuclear Factor-κ B Ligand (RANKL) and prevent its interaction with RANK to inhibit bone resorption, is also a potential therapeutic drug for myeloma bone disease 7, 8. Although the effectiveness of denosumab and zoledronic acid is comparable in specific populations, denosumab is less nephrotoxic in terms of adverse effects. Moreover, denosumab may even have a survival advantage over zoledronic acid in some subgroups 9-11. Based on the side effects and cost of these two drugs, MMBD therapies with fewer adverse events are urgently needed clinically.

In recent years, many studies have found a non-negligible association between gut microbes and various diseases, such as colorectal cancer and liver cirrhosis. Gut microbes play a crucial role in the occurrence and progression of tumors as well as the efficacy of immunotherapy 12. Studies have shown that compared with healthy matched controls, MM patients have significantly different gastrointestinal microbial composition, and some bacteria, such as *Clostridium prazines*, were significantly correlated with the International Staging System (ISS) stage 13. Moreover, patients with minimal residual disease (MRD)-negative status have a higher relative abundance of *Eubacterium hallii* than MRD-positive patients 14. In addition, the reduction of periodontal disease and oral biodiversity was also accompanied by a higher incidence of osteonecrosis of the jaw (ONJ) ​​in the treatment of MMBD treated with higher bisphosphonate concentrations. Further mechanistic investigations in mice revealed that the bacterial diversity in MM is higher, especially nitrogen-cycling bacteria, which can promote the progression of MM through circulating urea nitrogen 15. Furthermore, *Prevotella lysosides* in the gut flora can promote the differentiation of T helper 17 cells (Th17) cells, which migrate to the BM and further contribute to the progression of MM 16. The above studies have primarily focused on MM progression; however, whether the gut microbiota influences MMBD has not been studied before.

Studies have shown that the gut microbiota plays a role in the development of various bone diseases. By comparing the gut microbiota, procollagen type I N-terminal peptide (PINP), and other related factors between healthy controls and osteoporosis patients, it has been shown that the diversity of gut microbiota in patients with osteoporosis is reduced and imbalanced; these include the *Lactobacillus acidophilus* and *Lactobacillus rhamnosus* supernatants which can stimulate the proliferation, differentiation, and maturation of osteoblasts 17. Compared with conventionally reared mice, germ-free (GF) mice exhibited increased bone mass and decreased bone surface osteoclasts, accompanied by reduced CD4^+^ T cells and OC precursor cells in the BM and increased levels of tumor necrosis factor α (TNFα) 18. Several studies yielded similar results and found that the commensal microbiota may prevent hypercalcification by stimulating osteocalcin expression in osteoblasts and enhancing osteoblasts and osteoclasts via specific transcription factors 19. In terms of mechanism, studies have shown that segmented filamentous bacteria (SFB) can induce TH17/IL17A immunity, which has catabolic/anti-anabolic effects in bone 20. The rat obesity-insulin resistance model established by high-fat feeding also showed changes in bone tissue morphology, manifested as reduced trabecular thickness, increased osteoclasts and active eroded surfaces, as well as significantly reduced mineral deposition and bone formation rates; while probiotics and prebiotic therapy can improve metabolic disorders and osteoclast-related bone resorption and enhance osteogenic activity 21-23. Also, interleukin-17 (IL-17) and Th17 has been shown tumor-promoting effect interleukin-17A producing T helper 17 cells are significantly elevated in blood and BM in multiple myeloma and IL-17A promotes MM cell growth via the expression of IL-17 receptor. Anti-human IL-17A human monoclonal antibody (mAb) has been observed significant inhibition of MM cell growth. In a mice model, it leds to a significant inhibition of tumor growth and reduced bone damage compared with isotype control mice 24. In conclusion, the gut microbiota influences bone formation and the occurrence of various bone diseases via multiple mechanisms, but its impact on MMBD and how it works remains elusive.

To that end, the present study investigated the role of gut microbiota in MMBD and its related mechanisms. Antibiotics (ABX) induced dysbiosis has been shown to alter the composition and metabolism of gut microbiota 25, which may impact bone disease progression in MM patients. Herein, a mouse model with gut microbiota decimation induced by mixed antibiotics and a mouse model of myeloma bone disease induced by intravenous injection of mouse-derived myeloma Merwin Plasma Cell tumor-11 (MPC-11) cells were established. Bone changes were detected using micro-CT, while possible mechanisms were explored using gut microbiota 16S rRNA sequencing. Flow cytometry were used to deteted BM tumor load and Th17 cell. We show that the gut microbiota plays an important role in both MM and MMBD, the trend was accompanied by similar changes in serum IL-17 concentration and spleen Th17 cell count.

## Materials and Methods

### Cells, cell culture, and mice

The BALB/c-derived MPC-11 mice myeloma cell lines were kindly donated by professor Wang Siqing of The First Hospital of Jilin University. Cells were incubated in Roswell Park Memorial Institute (RPMI) 1640 medium (Gibco) supplemented with heat-inactivated fetal bovine serum (10%), L-glutamine (2mM), and penicillin/streptomycin 26. Female BALB/c mice were purchased from Lanzhou Institute of Veterinary Medicine, Chinese for Academy of Agricultural Sciences and housed in Lanzhou University Medical Experiment Center SPF Standardized Animal Laboratory. Mice were housed in micro isolators, under a 12 h/12 h dark/light cycle. Water and food (regular chow diets) were available ad libitum. Cage of mice were numbered and divided into different groups by random number sequence for a baseline value. 4-6 mice per group as shown in other paper. Computer-generated random numbers were used to randomize mice. To construct a model depleted of intestinal flora, 4 weeks old mice were started on an antibiotic mixture (ABX) of ciprofloxacin (100 µg/ml) and metronidazole (250 µg/ml) that was dissolved in drinking water 27, while the litter control mice were just treated with sterile water. To establish the multiple myeloma mice model, 1-5 × 10^6^ MPC11 cells were injected into the tail-vein of indicated mice. The control group and ABX group were injected RPMI 1640 medium only as control. Each group included 4 to 6 mice, and total about 20 mice were used as described in previous report 28. For bone analyses, mice were sacrificed using overdose of anesthesia before paralysis appeared after 4 weeks of tumor bearing. For the multiple myeloma mice with deficient gut microbiota models, mouse-derived myeloma cells were injected 4 weeks following antibiotic treatment. Observers did not know the specific grouping of each animal. Cages for all experimental mice were placed on the same floor shelf in the same room. All experimental manipulations were completed within 2 hours for a single experiment. All animal experiments were approved by the ethical review board of laboratory animal welfare at the Second Hospital of Lanzhou University (Approval reference number: D2021-322). All methods were performed in accordance with the relevant guidelines and regulations.

### Fecal microbiota transplantation (FMT)

Fecal microbiota transplantation was performed according to the previous paper 29. In short, 4-6-week-old male Balb/C mice received antibiotics (described above) for 4 weeks to delete the gut microbiota. After that, ABX continuous mouse feces were collected and transplanted every two days to withdraw the ABX operation but maintain the bacterial flora changes caused by it. The cecal content of the donor mice (ABX group) was collected and resuspended in PBS at 0.125g/ml. An amount of 0.15 ml was administered to mice by gavage at FMT group.

### 16S rRNA Sequencing of fecal sample

**DNA extraction and amplification:** Caecal samples were snap-frozen and stored at -80 °C after collection. Bacterial DNA was isolated from the samples using a MagPure Soil DNA LQ Kit (Magen, Guangdong, China) following the manufacturer's instructions. DNA concentration was measured using a NanoDrop 2000 spectrophotometer (Thermo Fisher Scientific, Waltham, MA, USA), while integrity was measured using agarose gel electrophoresis. The V3-V4 hypervariable region of the bacterial 16S rRNA gene was amplified by PCR using universal primers (343F: 5'-TACGGRAGGCAG-3'; 798R: 5'-AGGGTATCTAATCCT-3'). Sequencing results were linked to Illumina sequencing adapters by barcodes contained in reverse primers.

**Library construction and sequencing:** Amplicon quality visualization was measured using gel electrophoresis. We purified PCR products with Agencourt AMPure XP beads (Beckman Coulter Co., CA, USA) and quantified with Qubit dsDNA assay kit. DNA concentration was adjusted before sequencing. Sequencing was implemented using Illumina NovaSeq6000(Illumina Inc., San Diego, CA). Two paired-end read cycles of 250 bases each.

**Bioinformatic analysis:** Paired-end reads were preprocessed using Trimmomatic software to detect and cut off ambiguous bases(N) 30. Low-quality sequences with an average quality score below 20 were also removed using a sliding window trimming approach as reported previously by others 31. After trimming, paired-end reads were assembled with the FLASH software 32. Assembly parameters were a minimum overlap of 10 bp, a maximum overlap of 200 bp, and a maximum mismatch rate of 20%. Sequences were denoised by discarding reads with ambiguous, homologous sequences or below 200 bp as reported in previous articles 33. Reads with 75% of bases above Q20 were retained using QIIME software (version 1.8.0) 34, while those with chimera were detected and removed with VSEARCH software 35. Primer sequence removal and clustering of clean reads using VSEARCH software generated operational taxonomic units (OTUs) with a similarity cut-off of 97% 35. The representative read of each OTU was selected using QIIME package 36. All representative reads were annotated and blasted against the Silva database (version 132) using the RDP classifier (at a confidence threshold of 70%) as shown previous study 37. The microbial diversity in caecal content samples was estimated using the alpha diversity that accessed by Shannon index 38. The UniFrac distance matrix performed by QIIME software was used for Principal Component Analysis (PCA). The 16S rRNA gene amplicon sequencing and analysis were conducted by OE Biotech Co., Ltd. (Shanghai, China). Pathway enrichment analysis was performed using Kyoto Encyclopedia of Genes and Genomes (KEGG) and database by PICRUSt1 39, 40.

### Bone micro-CT (μ-CT)

At the end of the experiment, the mice were carbon dioxide anesthesia euthanized and their left femurs were taken, and the surface muscle tissue was removed as much as possible. Put the prepared tissue sample into 4% paraformaldehyde solution and fix it for 24-48h. Then remove the sample and wash it three times with phosphate buffered saline (PBS) buffer, and put the sample in a 75% alcohol solution at 4°C for later use. The fixed tissue samples were taken from the fixative, dry the fixative on the surface of the specimen, wrap the samples with plastic film, and use the SkyScan 1176 small animal Micro-CT scanning imaging system of Bruker, Germany, to scan the samples. The scanning parameters are set to: scanning resolution 18 μm, scanning voltage 50 kV, scanning current 500 μA, exposure time 225 ms. The image reconstruction software is NRecon software (Version: 1.7.4.6), and the microstructural parameters of the bone tissue in the target area are analyzed by CTAn software (Version: 3.0.0): 1.15.2.2). All the analysis range of all specimens is consistent.

### Spleen and bone histological analyses and immunohistochemical staining

Spleens obtained from mice were fixed in 4% paraformaldehyde in PBS (4% PFA/PBS). After blocking with paraffin, the fixed tissues were sliced into 2 μm sections and histologically examined following hematoxylin and eosin (HE) staining or immunohistochemical staining using anti-Syndecan-1 (CD138; Abcam, ab128936).

The femurs were fixed in 4% paraformaldehyde solution in 0.1 M PBS for 24 h at 4°C and then washed using PBS and decalcified in 10% EDTA for 4 weeks. Following a graded ethanol series dehydration, the samples were cleared in xylene, embedded in paraffin, and incised into thin sections (4um) in coronal. After been deparaffinized, the serial sections were stained with hematoxylin and eosin (H&E), TRAP or immunohistochemical staining of CD138 and RANKL (proteintech, 66610-1-Ig).

### ELISA

The serum bone resorption marker CTX-1 (Cat. # m1002251-2), the serum bone formation marker P1NP (Cat. #1063063-1) were measured using ELISA kits from Shanghai MLBLO Biotechnology Co.Ltd according to the manufacturer's instruction.

### Flow cytometry

All experments were performe as as described 41. CD138 surface staining was performed with PE anti-mouse CD138 (Syndecan-1) Antibody (Biolegend, 102503). Th17 cell staining was performed with Rat Anti-Mouse CD4 (RM4-5) PerCP-Cyanine5.5 (Biolegend, 561115); IL-17A Monoclonal Antibody (eBioscience, eBio17B7), APC(17-7177-81) and FITC anti-mouse CD3(17A2) Antibody(Biolegend, 100204). Cell Stimulation Cocktail (500X) (00-4970-93) and Intracellular Fixation Permeabilization Buffer Set (88-8824-00) were purchased from eBioscience.

### Statistical analysis

FlowJo_V10 was used for flow cytometric analysis and ImageJ was used for immunohistochemical analysis. Statistical tests were implemented using Prism software (Graph Prism 5.0 Software Inc. CA, USA). The confidence interval is 95%. The data of weight are expressed as a mean ± standard deviation (SD) and the differences among the groups were evaluated by *t*-test. The Kruskal-Wallis test was used to analyse diversity of comparisons between groups. A value of *P*< 0.05 was considered statistically significant in the compared groups. No statistical methods were used to preordain sample sizes, but our sample sizes are similar to previous publications. Graphical abstract wasdrawn by Figdraw.

## Results

### Antibiotic treatment inhibit tumor burden in peripheral organ of myeloma mice model

**Figure 1:**
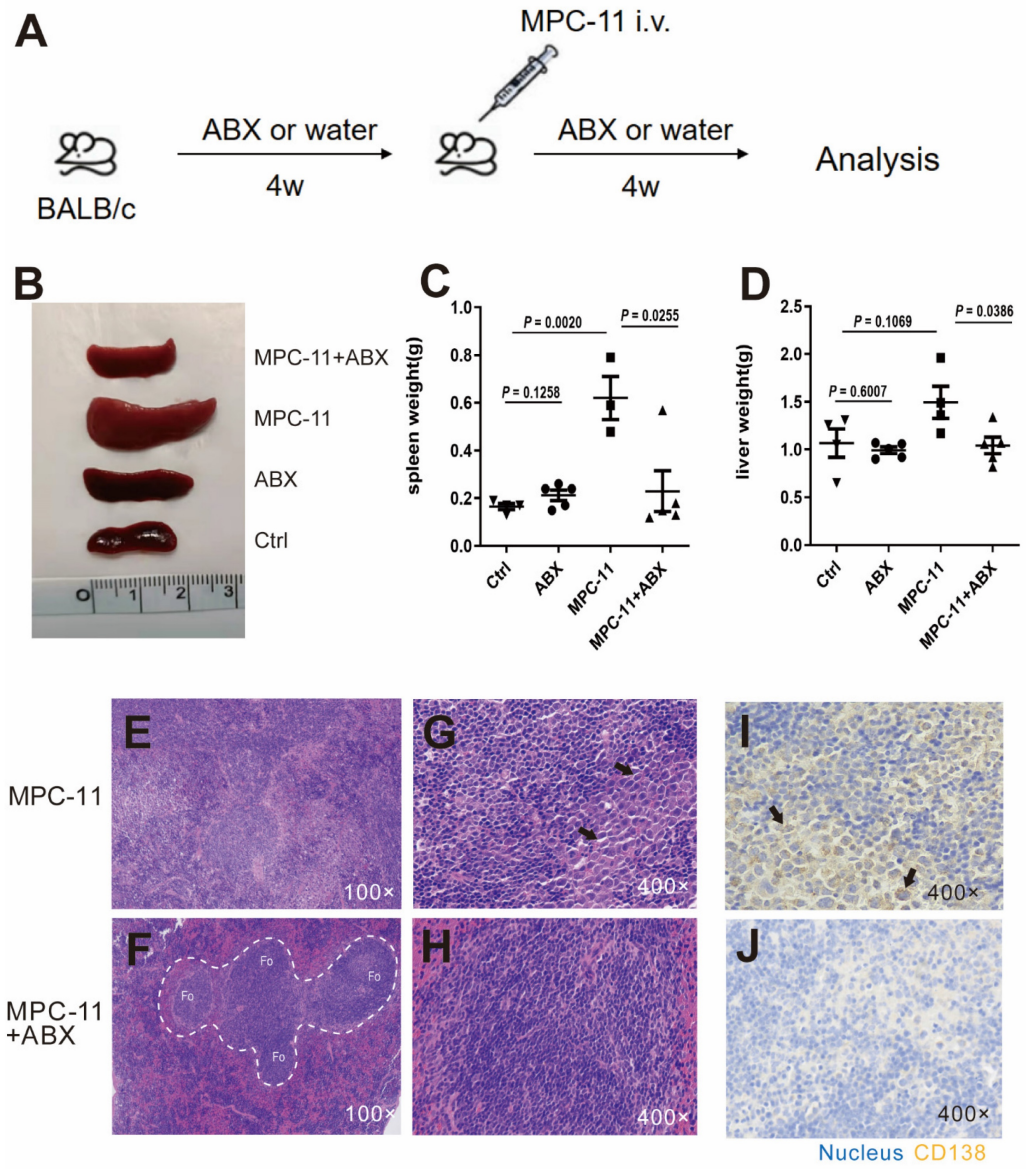
Intestinal flora deficiency inhibits tumor burden in peripheral organ of myeloma mice model. (A) Experimental procedure schematic. Four-week -old BALB/c mice raised under SPF conditions were treated with broad-spectrum antibiotics (ABX), and MPC11 cells were injected i.v. into BALB/c mice 4 weeks later. Analysis after another 4 weeks. N=4-5 for each group. (B) Spleen morphology of mice. Spleen(C) and liver(D) weight in each group. (E-H) Photomicrographs illustrating hematoxylin and eosin (HE) staining of spleen. (I and J) Immunohistochemistry (IHC) analysis of CD138 expression in spleen. Note: The white dotted line circled areas stand for follicular region, and the black arrows indicate plasma cells.

In order to construct a mouse model with intestinal flora deficiency, a mixture of antibiotics was fed 4 weeks in advance. Then MPC-11 cells were injected intravenously to mice in the intestinal flora deletion group and the control group to construct mouse myeloma models (Fig. 1A). The spleen and liver volume of the myeloma mice group was much larger than that of the mice without MPC-11 cells however antibiotic treatment reduced the enlargement of the spleen and liver (Fig. 1B-D). Using HE staining, we found that the spleen basic follicular structures formed by the T cell area surrounding the B cell area disappeared and replaced by diffusely distributed large cells with rich cytoplasm in myeloma mice (Fig. 1E-H). CD138, as a plasma cell-specific marker, immunohistochemical staining confirmed that the above large cells were derived from plasma cells. However, the mice treated with antibiotics showed a relatively normal spleen structure and rare CD138-positive cell infiltrating in the spleen (Fig. 1I and J). Because the Ctrl and ABX group just shown as normal, their data are presented in Supplementary Figure 1. In addition, in the absence of gut microbiota, the aforementioned indicators of anemia were reduced (Supplementary Figure 2). In summary, intestinal flora deficiency reduced the tumor burden in peripheral organ of myeloma mice.

### Antibiotic treatment inhibits tumor burden in bone marrow of myeloma mice model and not because of antibiotics but change of micaobiota

Because myeloma was mainly involved in bone marrow, we further detected myeloma load in bone marrow by flow staining marker CD138 in mouse femoral bone marrow. Here, in order to avoid the perious anti-tumor effect caused by ABX operation itself, we added intestinal flora transplantation experiment. That is, the antibiotic feeding was stopped 3 days before tumor bearing in ABX mice, and the bacteria flora of ABX mice were transplanted by gavage every other day to maintain a similar intestinal state, which was recorded as FMT-MPC11 group. Flow cytometry analysis showed that the levels of MPC11 mice and CD138-positive cells in the FMT group were higher than those in the CTRL group, while similar with which in the ABX MPC11 group.

### Antibiotic treatment alters the composition of the gut microbiota in mice

To evaluate alteration in the microbiota community structure between each group, the microbial alpha and β-diversity was measured as shown in Fig. 3A and 3B. This result indicates that alpha diversity of the gut microbiota in ABX groups was lower than that in control in terms of Shannon index. The principal component analysis (PCA) indicated the stool microbiome of ABX groups clustered significantly separately from that of the controls. But that between MPC-11 cells and non-MPC-11 cells were less different. Top10 boxplot of different species abundance was shown in Fig. 3C. In family level, as shown in figure, part of the microbiota was changed because of antibiotic treatment, and there was a big difference between the tumor bearing group and the non-tumor bearing group.

### Antibiotic treatment alleviated the bone damage in mice with myeloma

**Figure 2:**
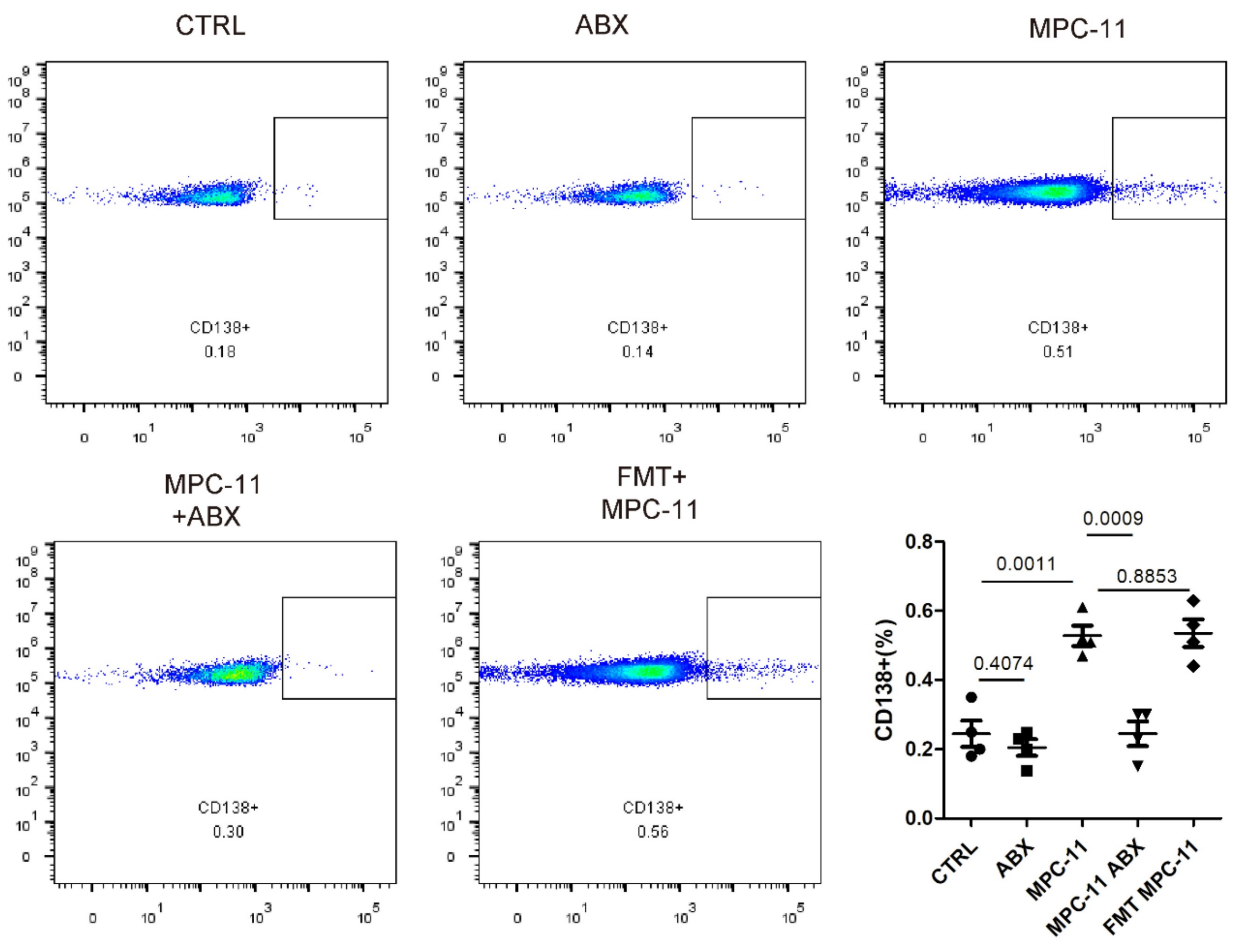
Intestinal flora deficiency inhibits tumor burden in bone marrow myeloma mice model. Analysis and statistical analysis of mouse femur by flow cytometry.

**Figure 3:**
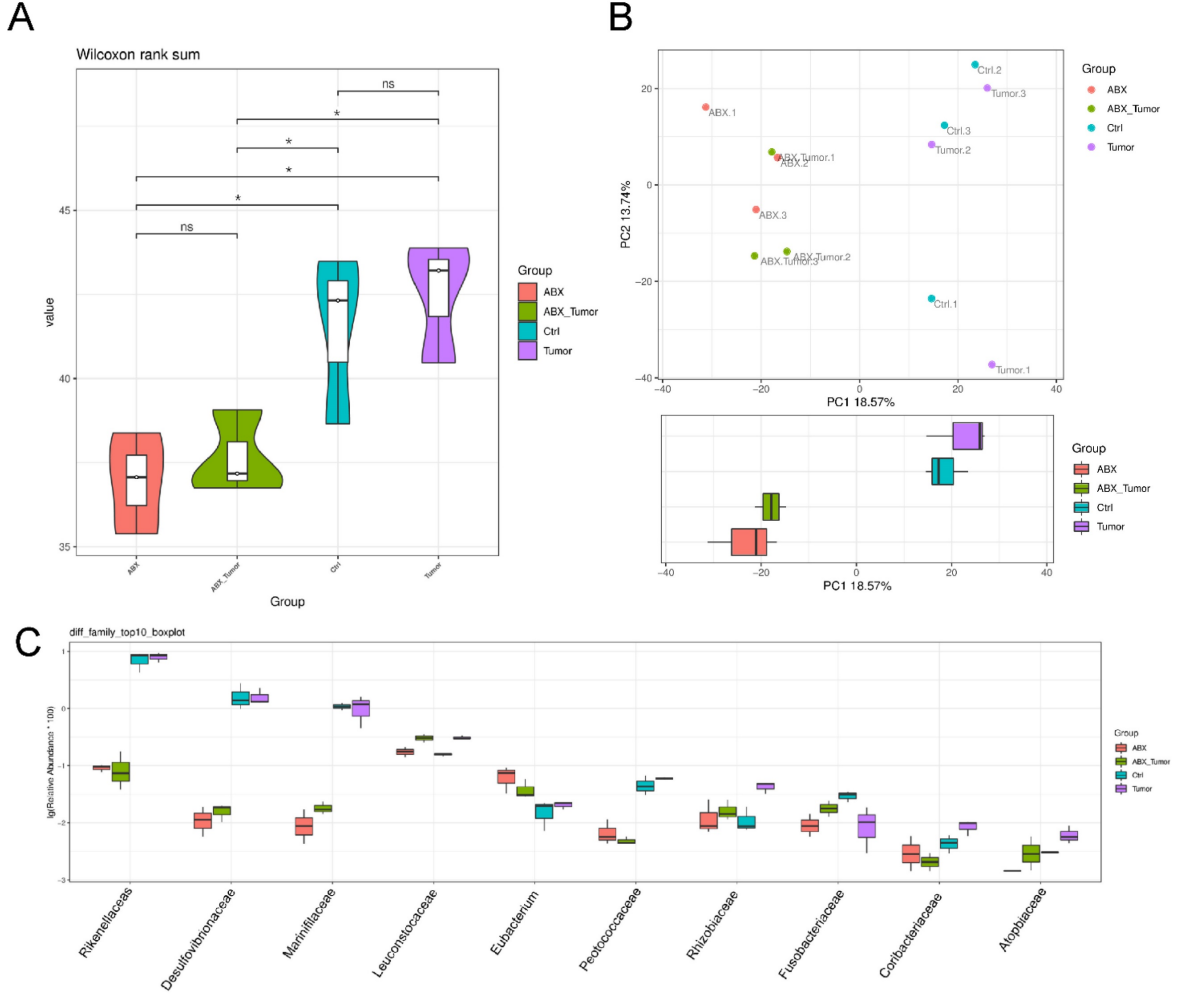
Analysis of 16S rRNA sequencing results. (A) The microbial α-diversity (as accessed by Shannon index) and (B) β-diversity (as accessed by PCA analysis). (C) Top10 boxplot of different species abundance. Note: n=3 for each group.

**Figure 4:**
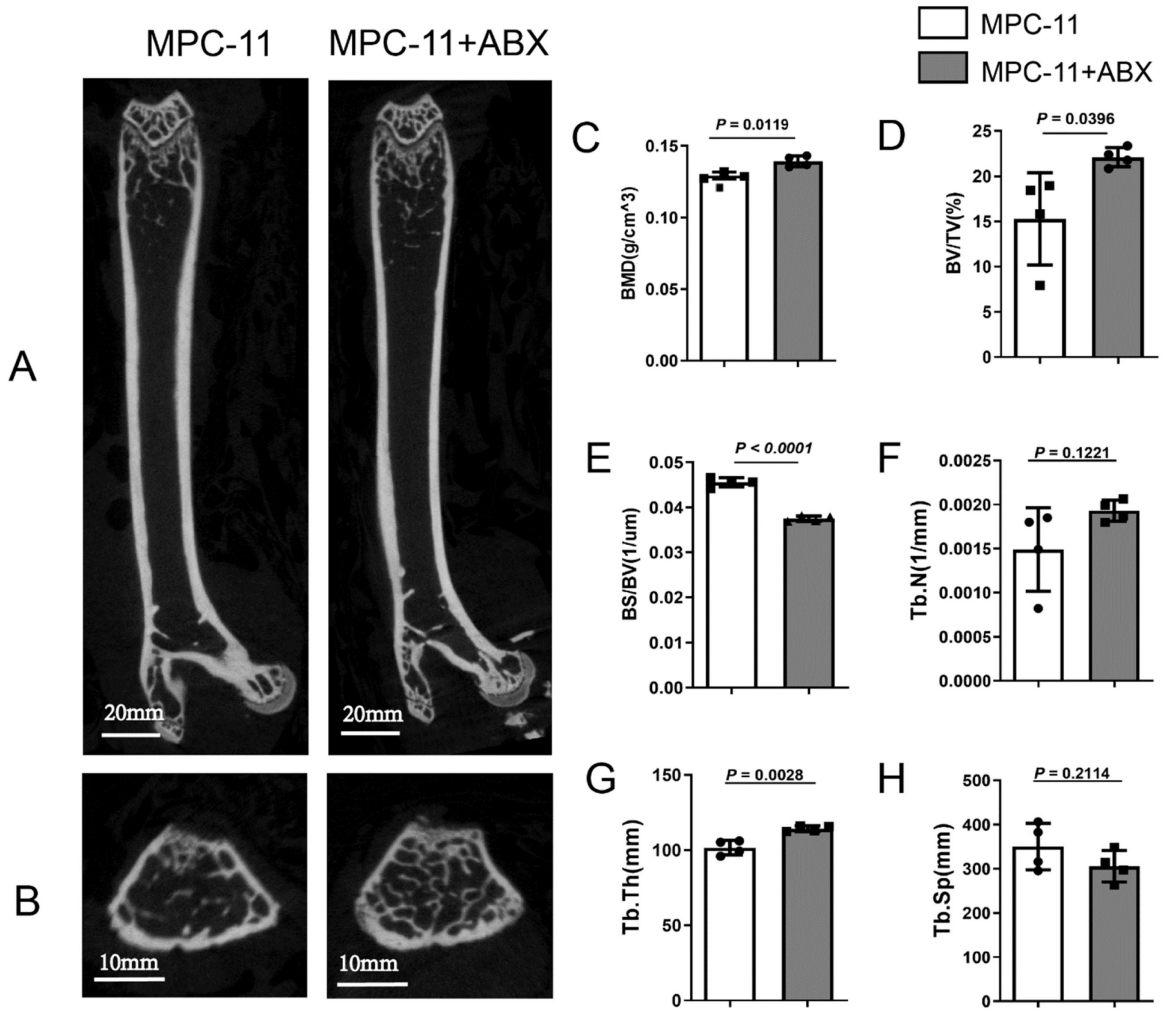
Intestinal flora deficiency impedes myeloma-mediated bone degeneration. Four weeks post MPC-11-bearing, the mice were killed, and formalin-fixed femur bones were imaged using μCT. Representative coronal(A) and transverse(B) image is shown (Scale bar, 600 μm.). Morphometric analysis of the bone mineral density (C), bone volume fraction (BV/TV) (D), Bone Surface/ Bone Volume (BS/BV) (E), Trabecular Number (Tb.N) (F), Trabecular Thickness (Tb.Th) (G), and Trabecular Separation/Spacing (Tb.sp) (H) in femoral shafts between Antibiotic-treated or not. Note: n=4 per group.

Due to the effects of intestinal flora changes on bone tissue reported in recent years, we also explored the effects of intestinal flora changes on bone of myeloma mice here. Compared with MPC11-BALB/c allograft mice without other treatments, those treated with ABX exhibited comparable higher bone trabecular network (Fig. 4A and B). After ABX treatment mice, the bone mass in myeloma mice was significantly increased, as well as the bone mineral density (BMD), bone volume fraction (BV/TV), trabecular bone thickness (Tb. Th) and Trabecular number (Tb.N); while trabecular separation (Tb.Sp) ratio of bone surface, width of medullary cavity between trabeculae, decreased (Fig. 2C-H).

### Intestinal flora deficiency myeloma mice have stronger bone mass

**Figure 5:**
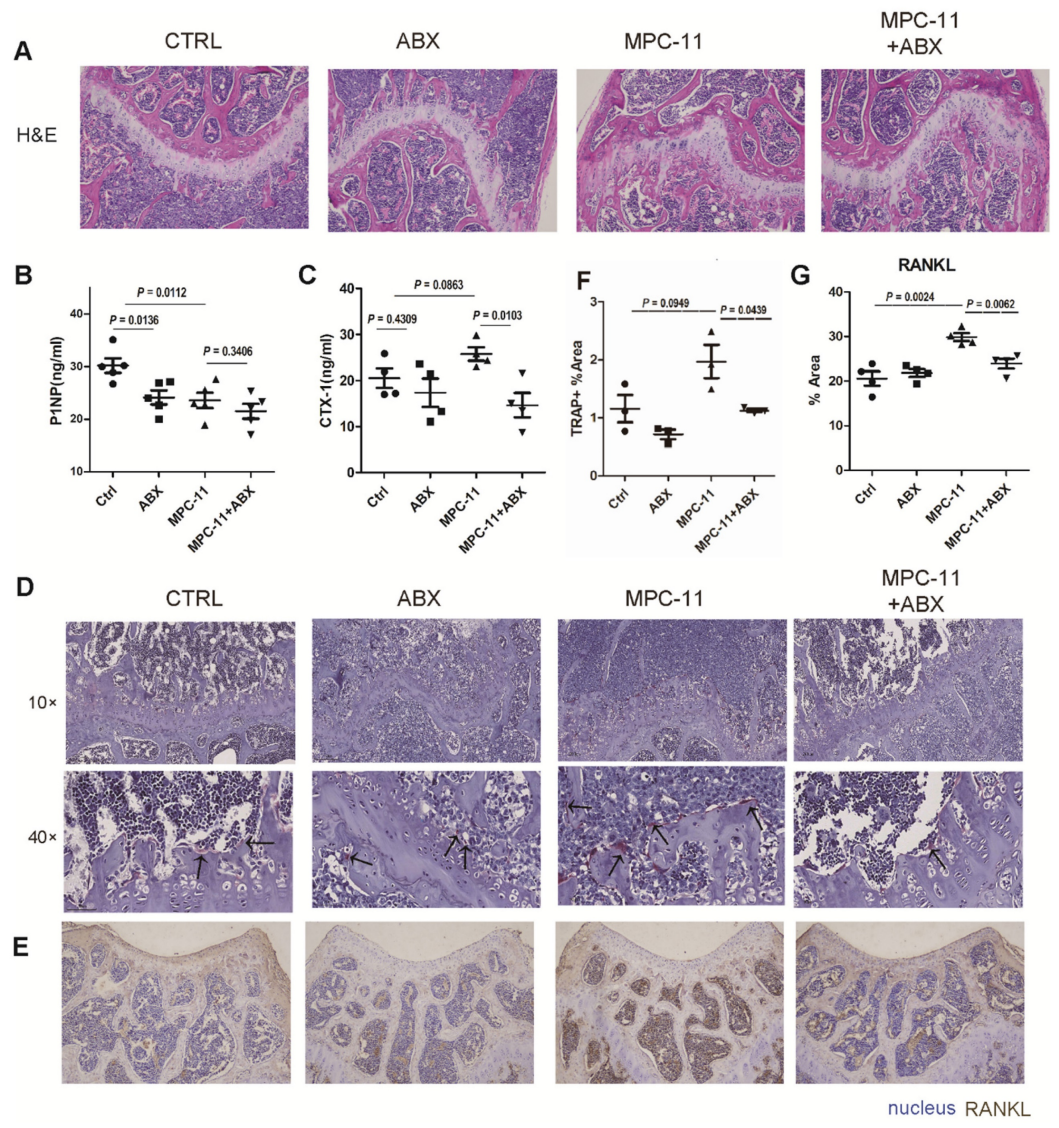
Intestinal flora deficiency myeloma mice have stronger bone mass. (A) Hematoxylin-eosin staining of mice left fumer in each group. (B and C) Serum bone resorption marker CTX-1 concentrations and serum bone formation marker P1NP concentrations in each group. (D) TRAP straining in each group. The positive cells appear dark wine red, as indicated by the black arrow. (E) RANKL immunohistochemical staining of mice left fumer in each group. RANKL-positive areas appeared dark brown in immunohistochemical staining. (F-G) Statistical analysis of Figure D and E. Note: n=3-4 per group.

To further illustrate the resulting changes in bone tissue, histological staining were performed on bone tissue after demineralization (Fig. 5A). By HE staining we found that the femoral plate chondrocytes of mice lacking intestinal flora were more than control group. Then, we examined some serologic markers of bone metabolism. The serum bone resorption marker CTX-I increased in MPC-11 bearing mice, however, it decreased when antibiotics mixture was given to eliminate gut microbiota (Fig. 5B). This suggests that antibiotic elimination of gut microbiota resulted in a reduction in bone resorption in myeloma mice. Interestingly, the serum bone formation marker P1NP were decreased in antibiotics treatment mice and lower in myeloma mice (Fig. 5C). To further confirm the bone resorption status of myeloma mice cells, TRAP staining and RANKL immunohistochemical staining were performed on bone tissue, which represent the resorption status. RANKL was significantly expressed in tumor-bearing mice, but decreased to baseline levels following antibiotic manipulation. In addition, we examined some serologic markers of bone metabolism. TRAP straining showed similar trand as RANKL. This suggests that antibiotic elimination of gut microbiota resulted in a reduction in bone resorption in myeloma mice. In conclusion, bone resorption was enhanced in tumor-bearing mice, and bone resorption was restored in myeloma mice after ABX manipulation.

### The reduction of myeloma tumor load due to ABX is accompanied by a decrease in Th17

**Figure 6:**
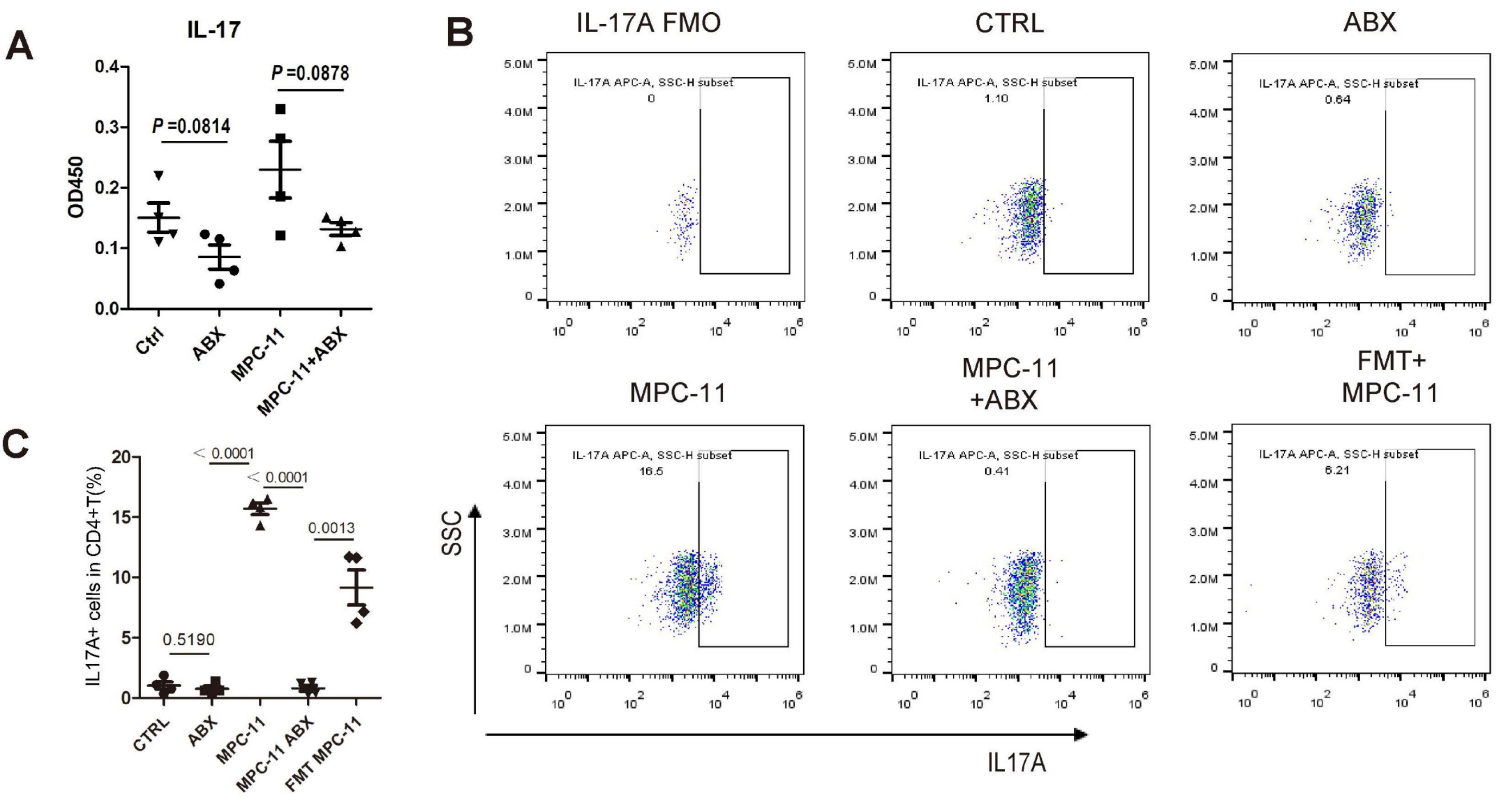
Intestinal flora deficiency myeloma mice have weakened Th17/IL-17 response. (A) Serum interleukin-17 concentration in mice. (B) Flow cytometric analysis of Th17 cells (IL-17A positive in CD3+CD4+T cells). (C) Frequency of IL-17A+CD3+CD4+ T cells in spleens of mice of each group. Note: n=4 per group.

In order to explore the reasons for the above phenomenon, IL-17 in peripheral blood of mice were detected (Fig. 6A). The results showed that the level of IL-17 in peripheral blood of mice increased after tumor bearing, but decreased after ABX. The number of Th17(CD3+CD4+Th17+) cells in the spleen of mice was further detected by flow cytometry, and the trend was consistent with the level of IL-17 and the frequency of Th17 increased after FMT (Fig. 6B and C).

## Discussion

To determine whether intestinal microbiota could affect bone degeneration in myeloma, we generated the syngeneic MPC11-BALB/c mouse allograft model. As demonstrated in other literature, the intravenous injection of MPC-11 in a model resulted in a reduction in red blood cell counts and hemoglobin levels in mice (Supplementary Figure 1), as well as a reduction in bone mass. This is a widely used and systematically mouse model of myeloma bone disease 26, 42, 43. We have shown that antibiotics-treated mice develop a much lower MM burden and consequently present better bone health according to micro-CT scan bone compared to the control counterparts, manifested as increased BMD, bone volume fraction, Tb.Th and Tb.N, as well as decreased Tb.Sp, which indicates increased bone resorption. Serological markers of bone metabolism showed that ABX decreased bone formation and bone destruction in tumor-bearing mice, but the overall effect was an increase in bone mass compared with tumor-bearing mice only. There are two possible reasons for the reduction in bone damage caused by ABX procedures. One is the reduction of MM load due to the loss of intestinal flora. In addition, ABX manipulation may also directly affect bone tissue metabolism through bone regulators (as seen in serum CTX-1 level and RANKL immunohistochemical staining). In multiple myeloma, because of the negative immunomodulatory effect of osteoclasts, bone destruction can further aggravate MM. In multiple myeloma, osteoclasts promote the formation of an immunosuppressive microenvironment for multiple myeloma, and thus bone destruction further exacerbates MM 44. Reduction in bone damage and reduction in myeloma were observed together, but it may be a chicken-and-egg question.

By 16S rRNA sequencing analysis, it was found the diversity of intestinal flora was significantly decreased after ABX. Previous studies have shown that fecal microbiota of multiple myeloma patients shifts compared to healthy controls 13, 45. They observed significant differences in the composition of bacteria in the gut between these two groups. The effect of intestinal flora on MM can be produced through various mediators. The MM-enriched bacteria showed higher abundance in MM patients with ISS-III than those of MM patients with ISS-II. Nitrogen-cycling microorganisms cause urea degradation, glutamine synthesis and absorption by the host, thus accelerating the tumor process of host MM 15. In addition, samples from MM patients included reduced numbers of Short Chain Fatty Acids (SCFAs)-producing bacteria that affect tumorigenesis of malignant plasma cell 13. Minimal residual disease (MRD) negativity in multiple myeloma is associated with higher *E hallii* abundance in their intestinal microbiota composition 14. Thus, it is possible that the composition of gut microbiota in MM patients has great influence on the effect of immunotherapy, especially taking into account that MM is closely related to immune response 46. Mechanistically, Microbial metabolites SCFAs can suppress proinflammatory cytokines IL-6 and TNF-α; whereas they may increase IL-10, Th17 and Th1 cells 47. Butyrate is one of the most crucial fatty acids and is associated with lots of biological process such as anti-inflammatory activities, cellular proliferation, inducing differentiation of regulatory T cells and apoptosis by activating several signal pathways such as Wnt/β-catenin and T cell receptor signaling 48, 49.

Therefore, the gut microbiota is expected to become a biomarker for multiple myeloma diagnosis, prognosis and treatment effect in the future. Furthermore, intestinal microbial transplantation is also expected to be applied to the treatment of multiple myeloma and the prevention of adverse events. Based on our study, it can be suggested that perturbation of the gut microbiota may also improve myeloma-related bone disease.

However, our study also has some limitations. As with most studies of gut microbiota, mice housed in different environments may have large differences in their gut microbiota. Even mice in the same environment of mice in different cages will have certain differences. Additionally, considering animal availability, we have not been able to validate this finding in more models, such as the 5TMM model, which may have limited generalizability. Antibiotic decimation as a method to eliminate microbiota has some limitations, which can be further investigated by different microbiota models such as germ-free mice 50.

## Conclusions

We found that the antibiotic feeding model can alleviate tumor burden in a systemic myeloma model, and for the first time found that this will further release myeloma-related bone disease. This study will provide new ideas for the current treatment of myeloma-related bone lesions in myeloma bone disease. It also reminds us to take into account the possible disturbance of the intestinal flora by drugs in the clinical treatment of myeloma, so as to provide better treatment for myeloma patients.

## Supplementary Material

Supplementary figures.
