# Supplementary material for: Gut microbiota deficiency ameliorates multiple myeloma and myeloma-related bone disease by Th17 cells in mice models
Source: J Cancer. 2023 Sep 25;14(17):3191–202. doi: 10.7150/jca.88799 (PMC10622987; doi:10.7150/jca.88799)
Supplement: Supplementary file 1 — Supplementary figures. [file jcav14p3191s1.pdf]

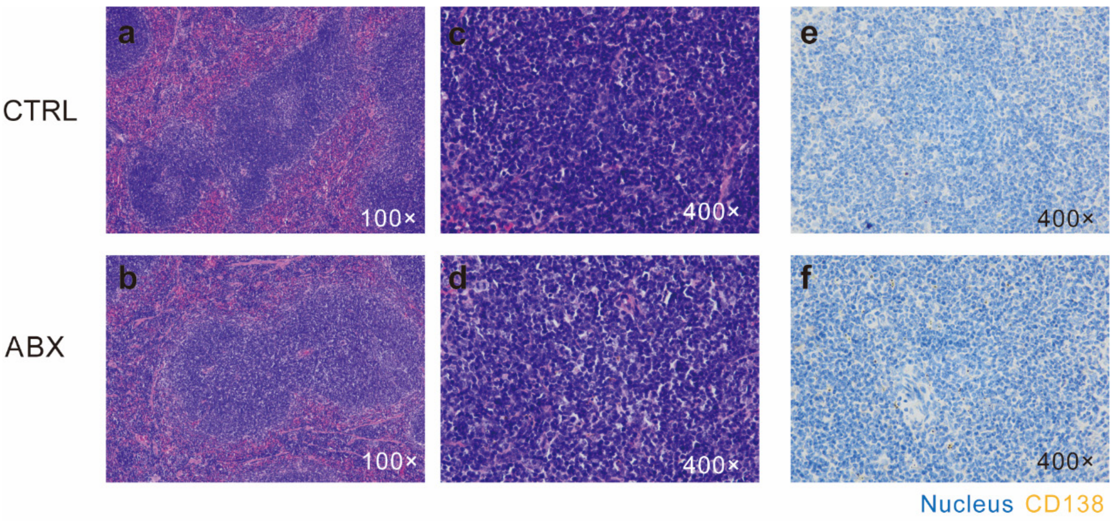

Supplementary Figure1. Morphological staining of Ctrl and ABX groups

(a-d) Photomicrographs illustrating hematoxylin and eosin (HE) staining of spleen of Ctrl and ABX group. (e and f) Immunohistochemistry analysis (IHC) of CD138 expression in spleen.

Note: The white dotted line circled areas stand for follicular region, and the black arrows indicate plasma cells.

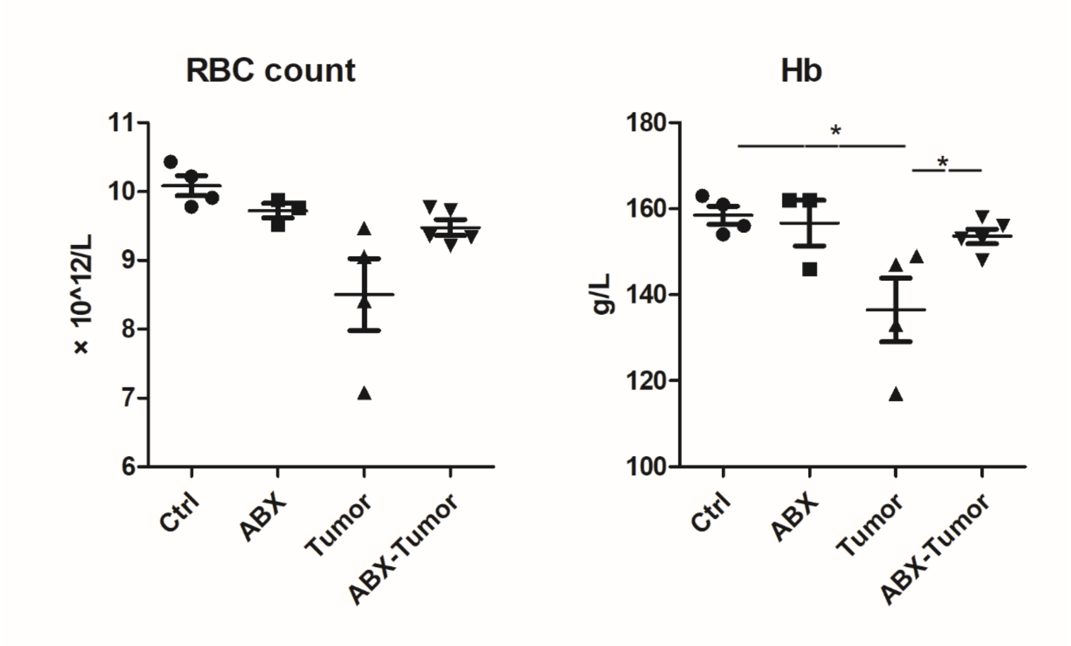

Supplementary Figure2. Peripheral blood red blood cell count and hemoglobin level in mice.

Note: n=3 in ABX group, n=4 in Ctrl and MPC-11 group, n=5 in MPC-11+ABX group.
